# Eukaryotic Initiation Factor 2α - a Downstream Effector of Mammalian Target of Rapamycin - Modulates DNA Repair and Cancer Response to Treatment

**DOI:** 10.1371/journal.pone.0077260

**Published:** 2013-10-25

**Authors:** Liron Tuval-Kochen, Shoshana Paglin, Gilmor Keshet, Yaniv Lerenthal, Charles Nakar, Tamar Golani, Amos Toren, Joachim Yahalom, Raphael Pfeffer, Yaacov Lawrence

**Affiliations:** 1 Cancer Research Center, Chaim Sheba Medical Center, Tel-Hashomer, Ramat-Gan, Israel,; 2 Sackler School of Medicine, Tel-Aviv University, Tel-Aviv, Israel,; 3 Department of Oncology, Memorial Sloan-Kettering, New-York, New York, United States of America,; 4 Department of Pediatric Hematology-Oncology, Safra Children's Hospital, Tel-Hashomer, Ramat-Gan, Israel; University of Missouri-Columbia, United States of America

## Abstract

In an effort to circumvent resistance to rapamycin – an mTOR inhibitor - we searched for novel rapamycin-downstream-targets that may be key players in the response of cancer cells to therapy. We found that rapamycin, at nM concentrations, increased phosphorylation of eukaryotic initiation factor (eIF) 2α in rapamycin-sensitive and estrogen-dependent MCF-7 cells, but had only a minimal effect on eIF2α phosphorylation in the rapamycin-insensitive triple-negative MDA-MB-231 cells. Addition of salubrinal – an inhibitor of eIF2α dephosphorylation – decreased expression of a surface marker associated with capacity for self renewal, increased senescence and induced clonogenic cell death, suggesting that excessive phosphorylation of eIF2α is detrimental to the cells' survival. Treating cells with salubrinal enhanced radiation-induced increase in eIF2α phosphorylation and clonogenic death and showed that irradiated cells are more sensitive to increased eIF2α phosphorylation than non-irradiated ones. Similar to salubrinal - the phosphomimetic eIF2α variant - S51D - increased sensitivity to radiation, and both abrogated radiation-induced increase in breast cancer type 1 susceptibility gene, thus implicating enhanced phosphorylation of eIF2α in modulation of DNA repair. Indeed, salubrinal inhibited non-homologous end joining as well as homologous recombination repair of double strand breaks that were induced by I-SceI in green fluorescent protein reporter plasmids. In addition to its effect on radiation, salubrinal enhanced eIF2α phosphorylation and clonogenic death in response to the histone deacetylase inhibitor – vorinostat. Finally, the catalytic competitive inhibitor of mTOR - Ku-0063794 - increased phosphorylation of eIF2α demonstrating further the involvement of mTOR activity in modulating eIF2α phosphorylation. These experiments suggest that excessive phosphorylation of eIF2α decreases survival of cancer cells; making eIF2α a worthy target for drug development, with the potential to enhance the cytotoxic effects of established anti-neoplastic therapies and circumvent resistance to rapalogues and possibly to other drugs that inhibit upstream components of the mTOR pathway.

## Introduction

The phosphatidylinositol 3-kinase - protein kinase B - mammalian target of rapamycin (PI3K-Akt-mTOR) pathway regulates cell growth and proliferation. The deregulation of the pathway underlies oncogenic transformations and its modulation by anti-neoplastic treatments affects their outcome. The mTOR's inhibitors - rapamycin, and its derivatives - decrease cancer cell proliferation and have been tested as anti-cancer agents in clinical trials [1–3]. Rapamycin has been used for coating stents to prevent angiographic-restenosis [4], and has won FDA approval as an immunosuppressant. Its derivatives - temsirolimous and everolimous have been approved for the treatment of various types of cancer [5,6].

Rapalogues bind their intracellular receptor FK506 binding protein 12 (FKBP12), forming a complex that inhibits mTOR complex 1 (mTORC1) by binding mTOR's FKBP12 rapamycin-binding domain [7]. Moreover, prolonged incubation with rapalogues can inhibit formation of mTOR complex 2 (mTORC2) [8]. However, the effect of rapalogues on mTORC1 and mTORC2 is cell type specific and may depend on the relative abundance of molecules that participate in the makeup of mTORC's macromolecular complexes [8,9]. Consequently the inhibitory outcome of rapalogues on tumor growth is not universal [7].

Therefore, in rapamycin-sensitive cancer cells, delineating rapamycin downstream effectors that modulate tumor growth and response to anti-neoplastic treatment is likely to lead to discovery of new compounds that will inhibit tumor growth and/or enhance its sensitivity to established therapies. Such molecules are expected to circumvent the resistance of cancer cells to drugs that target upstream components of the PI3K-Akt-mTOR pathway while having only a partial effect on its global activities. 

In the present study we report that inhibition of mTOR leads to increased phosphorylation of eIF2α - a subunit of eIF2. To date, contrasting reports have been published regarding the involvement of mTOR in eIF2α phosphorylation [10–16]. However, the present study demonstrates that in estrogen-dependent rapamycin-sensitive breast cancer MCF-7 cells as well as in triple negative rapamycin-insensitive MDA-MB-231 cells, inhibition of mTOR by rapamycin and by specific catalytic inhibitor (Ku-0063794) respectively, leads to increased phosphorylation of eIF2α. When bound to GTP, eIF2 recruits Met·tRNA^MET^ to the ribosome which then scans the capped mRNA. Following recognition of the initiation codon and GTP hydrolysis, the inactive eIF2·GDP is released and recycled into an active eIF2·GTP complex via interaction with the guanine nucleotide exchange factor eIF2B [17]. Under normal physiological conditions, eIF2α facilitates the interaction of eIF2 with eIF2B [18]. However, phosphorylation of eIF2α at its Ser^51^ turns eIF2 from a substrate of eIF2B into its competitive inhibitor, leading to a reduction in the level of eIF2·GTP·Met·tRNA^MET^ complex and to attenuation of global protein translation. Importantly, because the cellular level of eIF2 is in excess of eIF2B, a slight increase in eIF2α phosphorylation can sequester a major fraction of eIF2B [17]. In mammalian cells eIF2α is phosphorylated by four different kinases which respond differentially to various stress signals [17], and its dephosphorylation is conducted by the catalytic subunit of phospho-protein phosphatase 1 (PP1c) in complex with specific regulatory subunits e.g. the constitutive repressor of eIF2α phosphorylation (CreP) or the stress-induced growth arrest and DNA damage inducible protein (GADD34) [19]. Salubrinal – an inhibitor of eIF2α dephosphorylation - interferes with the association of PP1c and its regulatory subunits, thereby leading to increased eIF2α phosphorylation. Its application to various cell systems in vitro has been employed to elucidate the physiological relevance of increased eIF2α phosphorylation to cell survival [20,21]. 

The molecular outcome and physiological relevance of increased eIF2α phosphorylation has been extensively studied during endoplasmic reticulum (ER) overload where it is generally thought to exert a protective role. In response to increased ER load PKR-like ER-localized eIF2α kinase (PERK) is activated and a transient increase in eIF2α phosphorylation ensues [22]. This leads to a global attenuation of protein translation that may go hand in hand with increased translation of specific mRNAs that possess either an internal-ribosome-entry-site element [23] or short open reading frames in their 5’ leader [17]. The global attenuation of protein translation diminishes ER load, while specific proteins whose translation is increased activate transcription of genes that modulate cellular response to stress. The alleviation of eIF2α phosphorylation is required in order to enable the translation of the new transcriptome [24] 

Exposure of mouse embryonic fibroblasts (MEF) to anti-cancer agents - such as doxorubicin or histone deacetylase inhibitors - also led to increased phosphorylation of eIF2α, albeit a sustained rather than a transient one [25,26]. In these studies modified MEF carrying the non-phosphorylatable eIF2α A/A mutations showed higher sensitivity to the drugs than their S/S wild-type counterparts leading to the conclusion that the drug-induced increase in eIF2α phosphorylation is protective against cell death. However, while tightly regulated phosphorylation of eIF2α may be protective, an excessive and sustained eIF2α phosphorylation may be as harmful as its total abrogation. Indeed, it has been noted that while tightly regulated phosphorylation of eIF2α is required for proper embryonic development, a deficiency in phosphorylated eIF2α signaling, due to homozygosity for eIF2α A/A as well as excessive phosphorylation resulting from a knockout of CReP, a mutation that leads to inhibition of eIF2α dephosphorylation, are associated with fetal anemia and growth retardation [22]. Also, mutation in PERK and inhibition of eIF2α phosphorylation result in beta-cells death and Wolcott-Rallison syndrome, and similarly excessive eIF2α phosphorylation in TSC mutated cells following exposure to ER stressor inhibits expression of stress-induced transcriptome leading to cell death [24].

Our studies implicate sustained and excessive eIF2α phosphorylation in inhibition of DNA repair, development of senescence and decreased expression of a surface marker associated with capacity for self renewal, thereby providing a rationale for association with increased sensitivity to anti-neoplastic treatments such as ionizing radiation and histone deacetylase inhibitors (HDACi). Our results suggest that the development of drugs that increase eIF2α phosphorylation may provide additional means for enhancing the sensitivity to established anti-neoplastic therapies and/or circumventing resistance to drugs that target upstream components of the PI3K-Akt-mTOR pathway.

## Materials and Methods

### Cell culture

MCF-7 and MDA-MB-231 breast cancer cell lines, from American Type Culture Collection (Manassas, VA), were plated at a density of 4 ·10^3^ per cm^2^ and maintained as described before [27]. Cells were irradiated 48 hours post-plating in a Cs^137^ irradiator (Gammacell 1000 Elite/3000 Elan) at a dose rate of 475 cGy/minute or in X-ray irradiator (Polaris sc-500 series II) at a dose rate of 100 cGy/minute. Rapamycin, Vorinostat (LC laboratories, Boston, MA), Ku-0063794 (Selleck, Houston, TX) and salubrinal (Calbiochem-Merck4Biosciences, Germany) were added to the cultures from stock solutions in dimethyl sulfoxide (DMSO). Control cultures received equal amounts of the vehicle. DMSO concentration in the medium did not exceed 0.08%. Drugs were added to the culture immediately following radiation. 

### Colony survival assay

Plating for colony survival assay, colony counting, and calculation of surviving fractions was performed in triplicates as described before [28]. Unless otherwise noted, colonies were fixed, stained and counted 8-10 days following plating, when 90-95% of the colonies in control possess more than 50 cells. The theoretical additive effect, of combined anti-neoplastic treatments, on cell-survival was calculated according to the following formula: 100 x SFa x SFb (SFa = surviving fraction of cells treated with agent 'a'; SFb = surviving fraction of cells treated with agent 'b'). An experimentally determined surviving fraction that is lower than the calculated one indicates that the two agents have an enhanced rather than an additive inhibitory effect on cell survival. The underlying assumption of this equation is that the agents act independently of each other within the same population. The cellular fraction in percent that does not survive treatment (IF)% is equal to: [1-surviving fraction] x100 and the theoretical additive inhibitory effect of agents a and b on the size of killed cellular fraction in percent - (IFab)% is equal to [29]: 100 x [1-(1-Ia/100) x (1-Ib/100)] = 100 x (1-SFa x SFb). 

### Western blotting analysis

Preparation of cell lysates and analysis of treatment-induced changes in protein level and phosphorylation was done as described before [27] with minor modification. Protein content was determined with a bicinchoninic acid reagent (Bio-Rad, Hercules, CA), and equal loading was verified by measuring the absorbance at 520 nm of Ponceau S (Sigma, St-Louis, MO) extracted with PBS from individual strips of a twin run. Blots were exposed to x-ray films for chemiluminescence following treatment with West Pico ECL reagent (Thermo Scientific Rockford, IL). Values for integrated light density of autoradiograms were obtained with Image J NIH software and were employed for determination of treatment-induced changes in protein levels and in the ratio of phosphorylated eIF2α (p-eIF2α) to the total level of the protein. Rabbits antibodies that recognize either p-eIF2α or both the phosphorylated and non-phosphorylated eIF2α were from Cell Signaling Technologies (Boston, MA) and antibodies to BRCA1 (clone D9) and to CD2 were from Santa Cruz Biotechnology Inc. (Dallas, TX)

### Characterization of epithelial specific antigen (ESA) expression by FACS analysis

Control and salubrinal treated cells were detached with non-enzymatic cell dissociation solution (Sigma, Israel), incubated with human serum and FcR blocking reagent and then with fluorescent isothiocyanate (FITC)-anti-ESA or with isotype control (Miltenyi Biotec, Germany). 7-aminoactinomycin D (7AAD, eBiosciences, San Diego, CA) served as viability dye. Detection of cell staining was performed by FACSCalibur using Quest software (BD Biosciences, San Jose, CA) as described by Keshet et al. for staining of surface MDR1 [30]. 

### Staining for acidic β-Galacotosidase

Cell staining kit from Cell Signaling Technologies was used for cell staining and photomicrographs of stained cells were obtained with Nikon TS100 Eclipse inverted microscope and Nikon DS camera. 

### Assays for DNA repair

Non-homologous end joining (NHEJ) repair was assayed in HeLa cells and homologous recombination repair (HRR) was assayed in U2OS cells stably transfected with pEJSSA and pDR-GFP respectively [31,32]. The HeLa [33,34] cells were a gift from Dr. Dahm-Daphi, University of Hamburg and the U2OS were a gift from Dr. Scully, Harvard Medical School [32,34]. The assay was performed essentially as described by Moyal et al. and Seluanov et al. [34,35] except that cells were treated with 4.5 µM salubrinal 6 hours prior to transfection with plasmids expressing I-SceI (or empty vector in controls). For measurement of NHEJ, cells were co-transfected with I-SceI expressing vector and pDsRed2-N1 at a ratio of 10:1. Transfection was performed with LT1 DNA transfection reagent (Mirus Bio LLC.) according to manufacturer's instructions. Parallel, wild type GFP and DsRed expressing cells as well as negative controls were used for FACS calibration (adjusting voltage and color compensation). NHEJ activity was followed at the noted time post-transfection with I-SceI by monitoring GFP expression in DsRed expressing cells. Detection was performed by FACSCalibur at 50,000 events per sample. For determination of HR activity the fraction of I-SceI dependent GFP expressing cells was divided by the transfection efficiency in these cells as described by Moyal et al. [34]. Under our experimental conditions, the effect of salubrinal on the average fluorescence intensity in GFP expressing cells was always less than 10%, indicating that the noted effect of salubrinal on DNA repair does not result from inhibition of translation. Also, as shown in Table S1 in File S1, salubrinal did not alter the cell cycle distribution of U2OS cells indicating that the inhibitory effect of salubrinal on HRR activity following transfection with I-SceI results from direct inhibition of repair and not from an effect on the fraction of cells in S phase. 

### Transfection of cells with plasmids coding for eIF2α variants

heIF2α S51A and heIF2α S51D in pcDNA3.CD2 [36,37] were a gift from Dr. Ron's laboratory New York, NY. Transient transfection was carried out with JetPei (Polyplus, New York, NY) according to manufacturer's instruction. When transfection was performed in 10 cm culture dishes - 10 µg plasmids were incubated in 6 ml growth medium without antibiotics for 24 hours before adding 4 ml medium and proceeding with the experimental protocol. When transfection was carried out in 6 well dishes, the noted amounts of plasmids were incubated in 2 ml growth medium without antibiotics for 24 hours before adding 0.5 ml medium and proceeding with the experimental protocol. Expression of reporter protein was monitored in Western blots with anti-CD2. 

### Statistical analysis

Unpaired Student *t* test or one sample *t* test after logarithmic transformation was employed for statistical analysis. 

*p* < 0.05 was considered statistically significant.

## Results

It has been previously demonstrated that the estrogen dependent MCF-7 breast cancer cells are highly sensitive to rapamycin (IC_50_ - ~10 nM). In contrast, the triple negative MDA-MB-231 are insensitive to the drug (IC_50_ -5900 nM) [38] due to relatively high level of phosphatidic acid that competes with rapalogues for binding to the FRB domain [9]. Our results also show that while in MCF-7 cells rapamycin induces clonogenic death with an IC_50_ of ~50 nM, in the MDA-MB-231 cells concentrations up to 2 µM did not significantly affect cell survival (Table S2 in File S1). 

### eIF2α is a downstream target of rapamycin and irradiation

**Figure 1:**
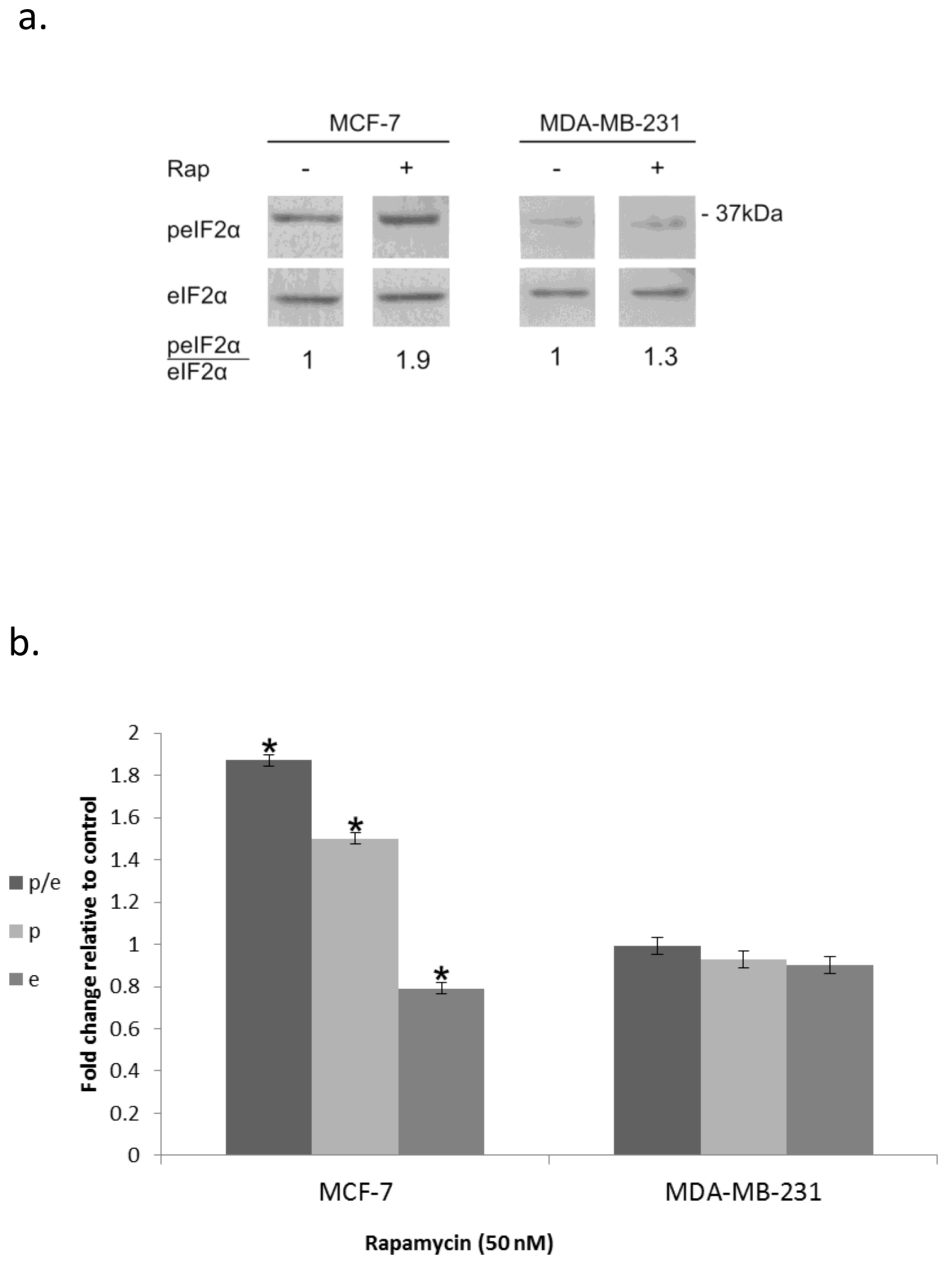
Increased eIF2α phosphorylation in response to rapamycin: **a**. Cells were incubated for 24 hours with 50 nM rapamycin, harvested and processed for determination of changes in eIF2α phosphorylation **b**. Mean ± SEM fold change relative to control of eIF2α (e), p-eIF2α (p) and the ratio p-eIF2α/eIF2α (p/e) in rapamycin treated cells. The analysis represents 6 separate determinations for MCF-7 cells and 3 for MDA-MB-231 cells. *Denotes significant change relative to control - *p*< 0.05.

**Figure 2:**
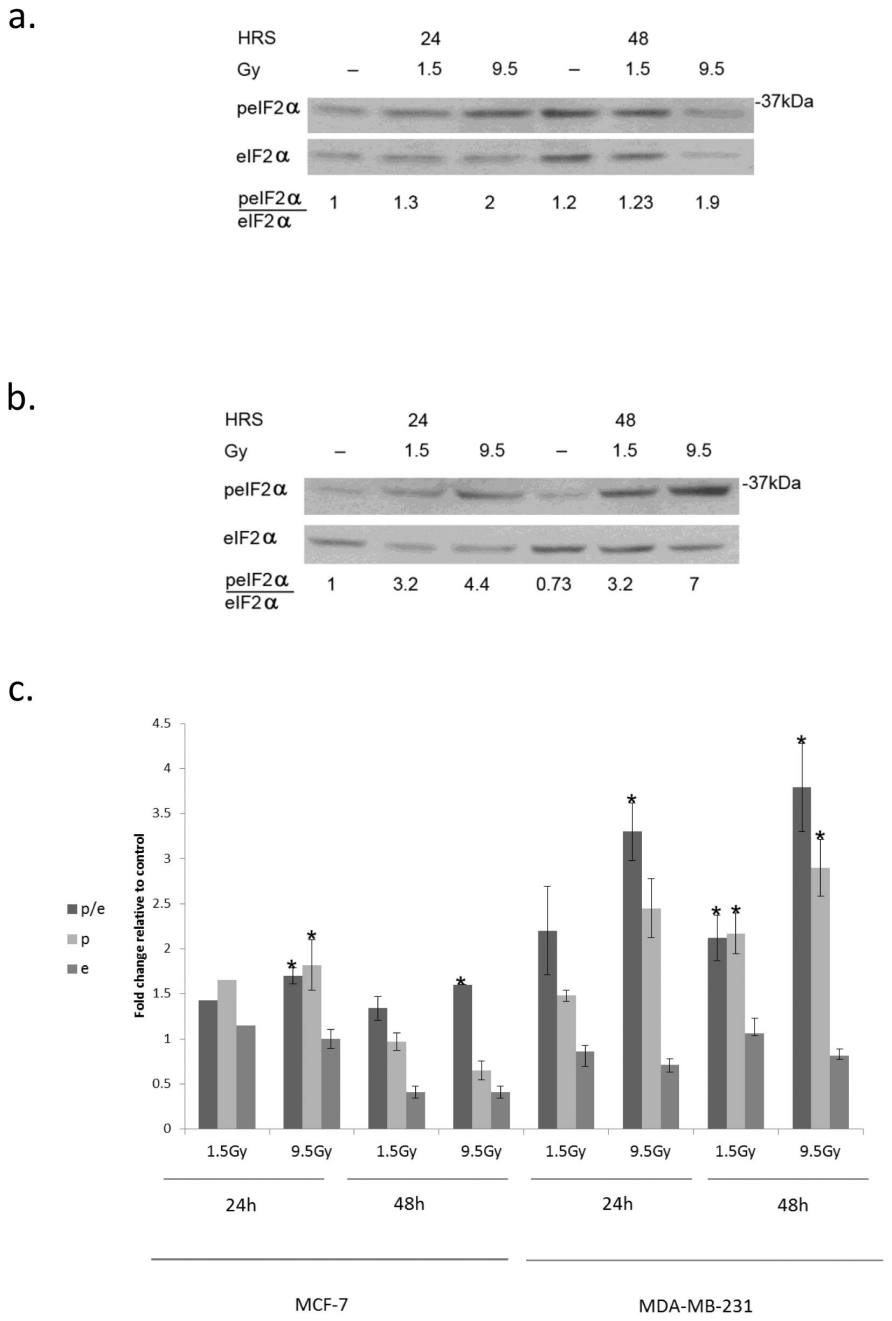
Increased eIF2α phosphorylation in response to radiation: **a**. MCF-7 and **b**. MDA-MB-231 cells were irradiated with the indicated radiation dose and processed for analysis of eIF2α phosphorylation at the noted time post-irradiation. **c**. Mean ± SEM of fold change relative to control of eIF2alpha (e), p-eIF2α (p) and the ratio of p-eIF2α/eIF2α (p/e) in irradiated MCF-7 and MDA-MB-231 cells. Analysis for MCF-7 at 24 hours post-irradiation with 1.5 and 9.5 Gy represents 2 and 3 separate determinations respectively and at 48 hours 3 separate determinations for each dose. Analysis for MDA-MB-231 cells at 24 hours represents 3 determinations for each dose and at 48 hours 5 determination for 1.5 Gy and 6 for 9.5 Gy. *Denotes significant change relative to control - *p*< 0.05.

At nM concentrations rapamycin led to increased phosphorylation of eIF2α which was much more pronounced in MCF-7 than in MDA-MB-231 (Figure 1 a-b). Ionizing radiation, on the other hand, increased eIF2α phosphorylation in both MCF-7 and MDA-MB-231 (Figure 2 a-c) cells. In MDA-MB-231 cells increased level of p-eIF2α as well as increased ratio of p-eIF2α to eIF2α was sustained through 48 hours post-irradiation. In some experiments a decrease in the total level of eIF2α in irradiated cells was noted, however this difference was not statistically significant. In MCF-7 cells the level of p-eIF2α as well as that of the total level of eIF2α decreased greatly by 48 hours following irradiation but the elevated ratio of p-eIF2α to eIF2α achieved by 24 hours post-irradiation was maintained. 

### Increased phosphorylation of eIF2α is detrimental to cell survival

**Figure 3:**
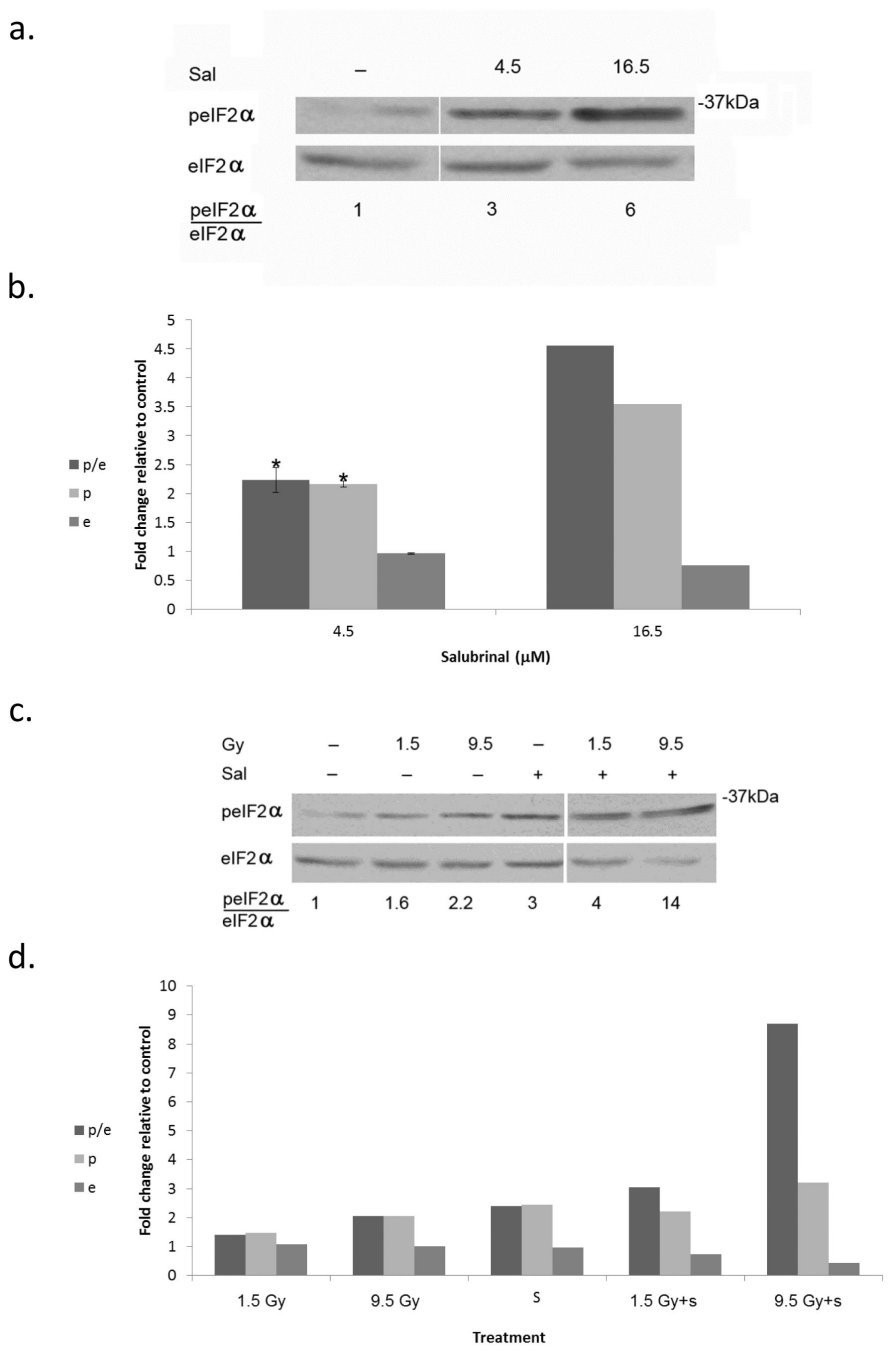
Salubrinal enhances radiation induced eIF2α phosphorylation: **a**. Cells were processed for analysis of eIF2α phosphorylation 48 hours post-addition of salubrinal (Sal). b. Mean ± SEM fold change relative to control of eIF2α (e), p-eIF2α (p) and of the ratio p-eIF2α/eIF2α (p/e) in salubrinal (Sal) treated cells. The experiment was reproduced 3 times for 4.5 µM and twice for 16 µM. * Denotes significant change relative to control - P<0.05. **c**. Cells were irradiated with the noted radiation dose prior to addition of 4.5 µM Salubrinal (Sal). **d**. Mean fold change relative to control of eIF2α (e), p-eIF2α (p) and the ratio p eIF2α/eIF2α (p/e).The experiment was reproduced once with similar results (Values for e, p, and p/e from both experiments are presented in Table S3 in File S1).

To determine the relevance of increased eIF2α phosphorylation to cell survival we treated MDA-MB-231 cells with salubrinal – an inhibitor of eIF2α dephosphorylation. Salubrinal led to a dose-dependent increase of eIF2α phosphorylation that was associated with increased clonogenic death (Figure 3 a,b; Table 1). Combining treatment of salubrinal - at a concentration that does not affect cell survival (4.5 µM) - and radiation led to increased phosphorylation of eIF2α that was associated with enhanced clonogenic death (Figure 3 c,d; Table 2; Table S3 in File S1). Interestingly, in cells that received combined treatment of 4.5 μM salubrinal and 1.5 Gy, phosphorylation of eIF2α was similar to that obtained in cells treated with salubrinal alone, suggesting that irradiated cells are more susceptible to increased eIF2α phosphorylation than non-irradiated cells. Enhanced sensitivity to radiation was also noted in salubrinal treated MCF-7 cells (Table S4 in File S1).

**Table 1:** Salubrinal induces dose-dependent clonogenic death.

**Salubrinal (µM)**	**IF (%)**
0	0±0.6
4.5	0±5
9	38±2
16.5	100

MDA-MB-231 cells were plated and processed for clonogenic assay. Numbers are IF(%) ± SEM of triplicate samples. The effect of salubrinal on cell death at 9 and 16.5 µM was significant (*p*<0.05).

**Table 2:** Salubrinal enhances clonogenic cell death in irradiated cells.

**Gy**	-**Sal IF** (**%**)	**+Sal IF (%)**	**Calculated Additive**
**0**	0±1	0±3	
**1**	3±0.3	8±2.6	3
**1.5**	17±0.6	30±1.6	17
**2.5**	48±1	66±2.6	48

MDA-MB-231 cells were plated for clonogenic survival assay. Salubrinal (Sal) (4.5 µM) was added to the cells immediately following irradiation. Numbers are means IF (%) ± SEM of triplicate samples from a representative experiment that was reproduced twice with similar results. Theoretical additive effect of salubrinal and radiation on the size of killed cellular fraction is presented under 'calculated additive'. The effect of salubrinal on increased clonogenic death within 1.5 Gy and 2.5 Gy radiation groups was significant (*p*<0.05).

**Figure 4:**
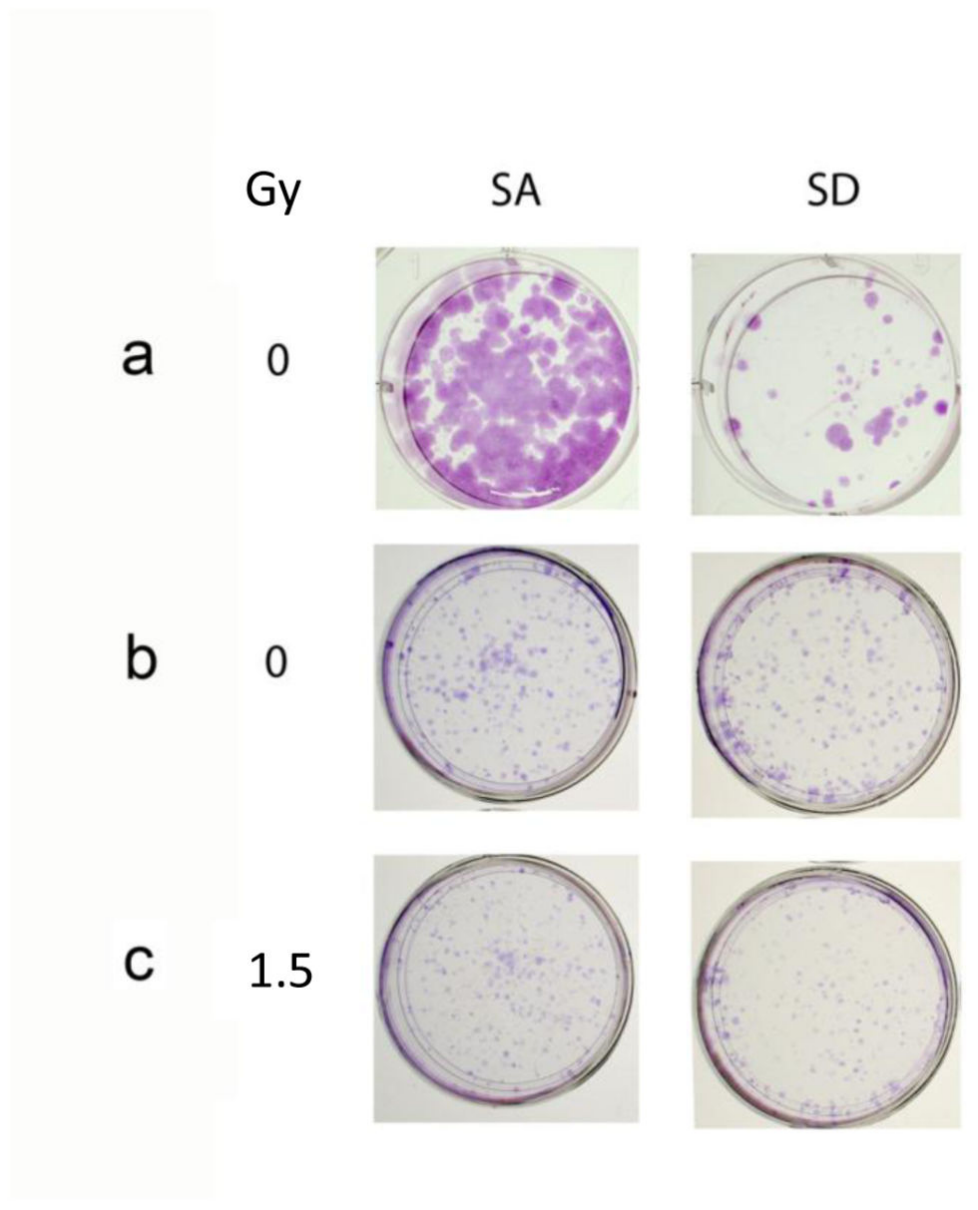
The phosphomimetic eIF2α variant increases clonogenic death in control and irradiated cells: **a**. Cells transfected with 2 µg/2ml eIF2α S51A (SA) or with eIF2α S51D (SD) were processed for analysis 17 days post-plating. At this concentration SD decreased clonogenic survival. **b**,**c**. Cells transfected with 1.5µg/2ml SA or with SD. Cells in **b** were not irradiated whereas cells in **c** were irradiated with 1.5 Gy 24 hours post-transfection. Cells in b and c were processed for analysis 10 days post-irradiation. At this concentration SD by itself did not affect clonogenic survival but did increase sensitivity to radiation. The experiment was reproduced once with similar results.

Similar to the effect of salubrinal, transient transfection with the phosphomimetic eIF2α S51D variant decreased - in a plasmid-dose-dependent manner - clonogenic survival relative to that observed in cells transfected with the non-phosphorylatable S51A variant. At high plasmid concentrations (Figure 4 a) survival of cells expressing the phosphomimetic variant was lower than that of cells expressing the non-phosphorylatable variant. However at a lower plasmid concentration eIF2α S51D did not affect survival relative to eIF2α S51A (Figure 4 b), but led to increased clonogenic death in irradiated cells (Figure 4 c, Table 3). Under our experimental conditions radiation-induced increase in the phosphorylation of endogenous eIF2α was similar in eIF2α S51A and eIF2α S51D expressing cells (Figure S1), indicating that similar to salubrinal the deleterious effect caused by expression of eIF2α S51D results from an increased cellular level of inhibited eIF2α i.e. eIF2α S51D and phosphorylated endogenous protein.

**Table 3:** The phosphomimetic eIF2α variant increases clonogenic death in irradiated cells.

**SA IF (%)**	**SA+1.5 Gy IF (%)**	**SD IF (%)**	**SD+1.5 Gy IF (%)**
0±3	48±0.5	0±4	62±1.5

Cells were transfected with 1.5µg/2ml eIF2α S51A or eIF2α S51D. Irradiation with 1.5 Gy took place 24 hours later. Colonies were processed for analysis 10 days post-irradiation. Numbers are means IF (%) from triplicate samples ± SEM. The experiment was reproduced once with similar results. Differences between control and irradiated groups as well as between the two groups of irradiated variants were significant *p*<0.05. SA - non-phosphorylatable eIF2α, S51A, SD - phosphomimetic eIF2α S51D.

### Salubrinal affects expression of surface ESA

**Figure 5:**
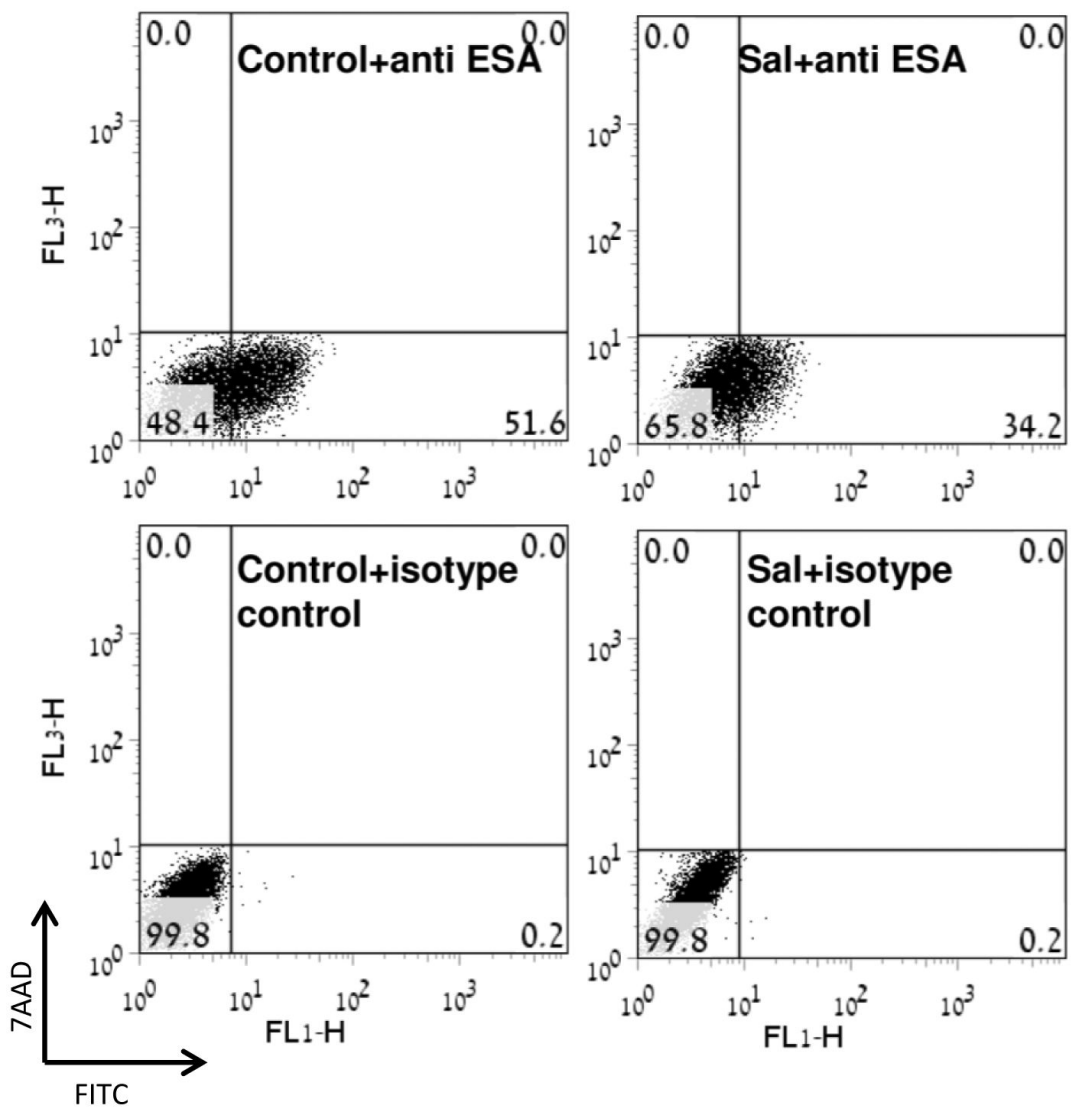
Salubrinal reduced expression of surface ESA: Cells were incubated with 24 µM salubrinal for 96 hours prior to harvesting, Cell staining with FITC-anti-ESA or with isotype-matched controls was detected with FACSCalibur. The experiment was reproduced twice with similar results. 7AAD - viability dye, FITC - anti-ESA.

It has been reported that tumorigenic breast cancer stem cells are enriched with a sub-population of cells expressing CD^44+^/CD^24^-/low/ESA^+^ on their surface [39], and that a sub-population expressing a similar combination of cell-surface markers has been identified in breast cancer cell lines (such as MDA-MB-231), and has been shown to possess high capacity for self renewal and tumor initiation [40]. Because over 90% of the MDA-MB-231 cells are CD^44+^/CD^24-/low^, sorting for cell surface ESA expression in these cells can serve as an indicator for changes in size of cellular fraction with self renewal capacity [40]. As noted in Figure 5, treating the cells with salubrinal decreased expression of ESA on cells' surface, suggesting that the deleterious effect of excessive eIF2α phosphorylation may diminish their capacity for self renewal. 

### Salubrinal induces senescence in breast cancer cells

**Figure 6:**
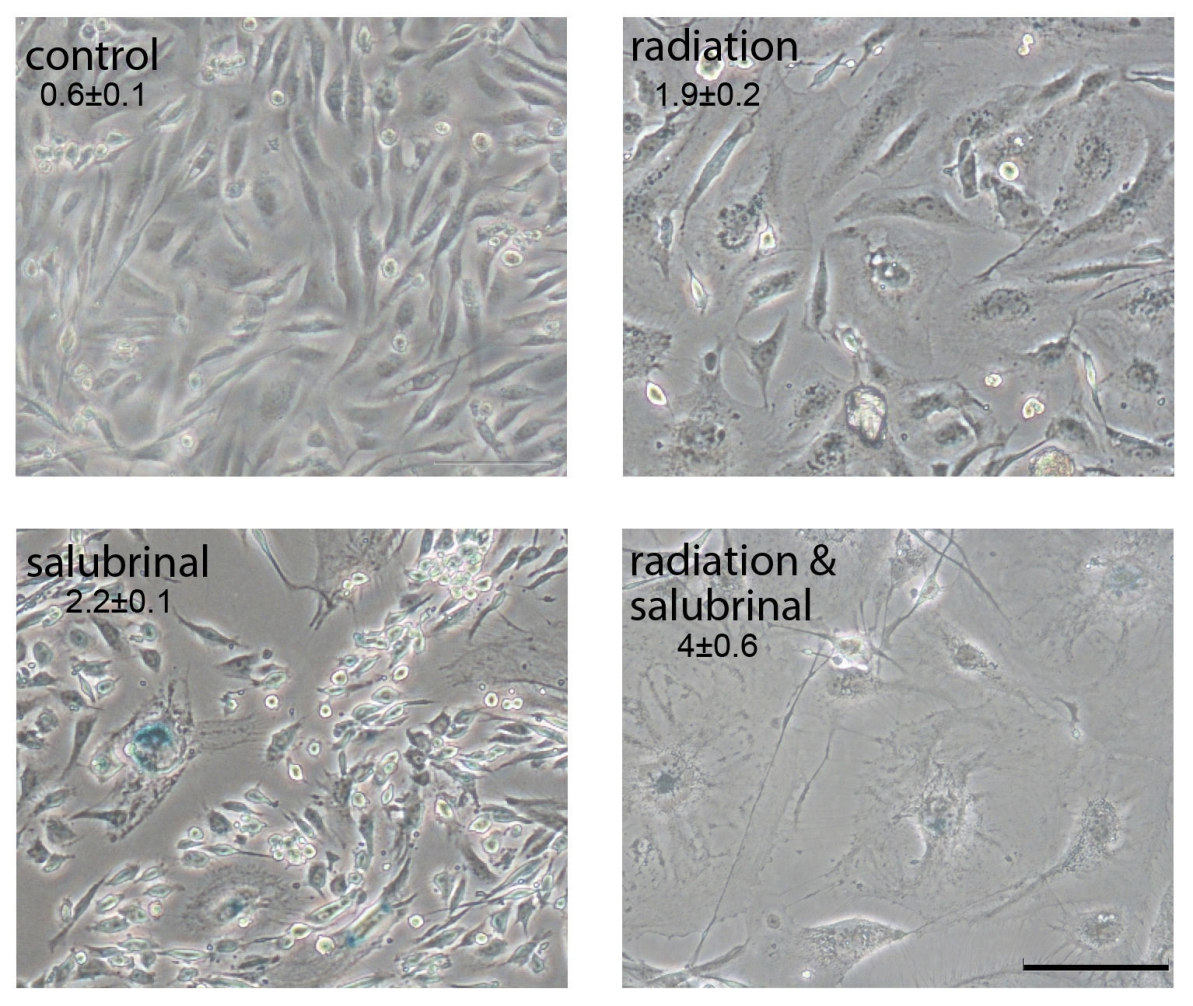
Salubrinal increases activity of senescence-associated β-Galactosidase: Cells were plated for colony survival assay. Salubrinal (4.5 µM) or the vehicle was added immediately following radiation with 1 Gy. Acidic β-Galactosidase activity is reflected in the blue stain. Numbers are average of senescent looking cells/colony ± SD in triplicate plates and differences between the combined treatments and each one of the sole treatments as well as between each treatment and control were statistically significant *p*< 0.05. Bar,100 µM.

Colonies of irradiated and salubrinal treated cells contain enlarged and senescent looking cells. We counted these cells in colonies formed following exposure to 1 Gy, to 4.5 µM salubrinal to the combination of radiation and salubrinal and in untreated controls. Interestingly, even though combined treatment of 4.5 μM salubrinal and 1 Gy did not lead to enhanced clonogenic death (Table 2) it did lead to enhanced appearance of senescent looking cells (Figure 6). Staining of acidic β-Galacotosidase showed that these large cells express the enzyme indicating that indeed salubrinal enhances senescence in irradiated cells (Figure 6). 

### Increased eIF2α phosphorylation modulates DNA repair

**Figure 7:**
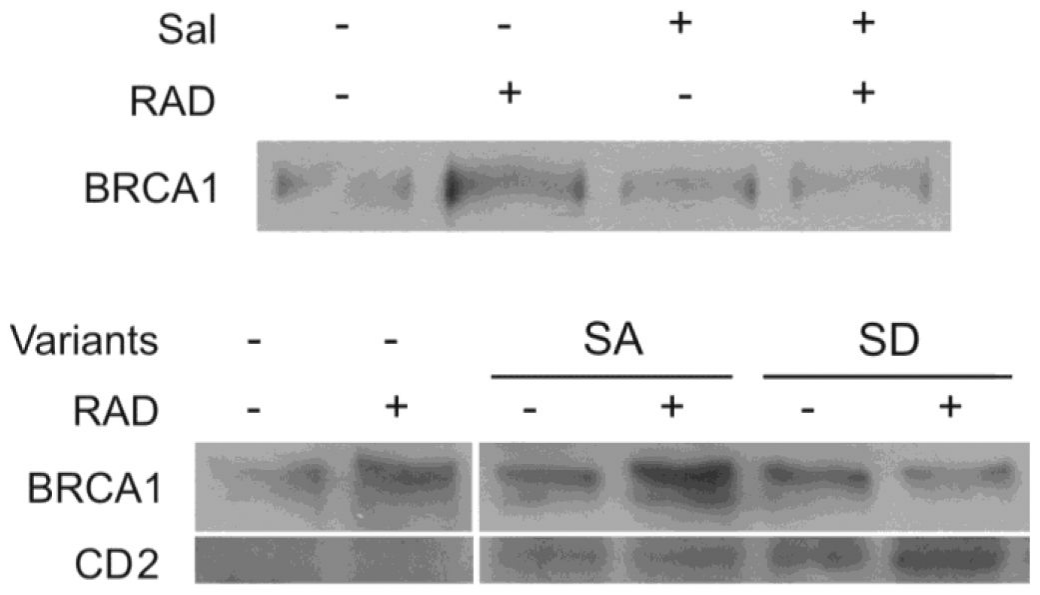
Increased eIF2α phosphorylation in irradiated cells abrogated radiation-induced increase in cellular BRCA1 Upper panel: Control and irradiated cells (1.5 Gy) were treated with 4.5 μM salubrinal or the vehicle. Cells were harvested 48 hours post-irradiation and processed for western blot analysis of changes in the level of BRCA1. Lower panels: Cells transfected with 10 µg/ml of the S51A and S51D eIF2α variants or with transfection reagents alone (SHAM). Cells were irradiated with 9.5 Gy 24 hours post-transfection and processed for BRCA1 analysis 24 hours post- radiation. CD2 was expressed in transfected cells indicating a successful transfection. Sal - 4.5 µM salubrinal.

**Figure 8:**
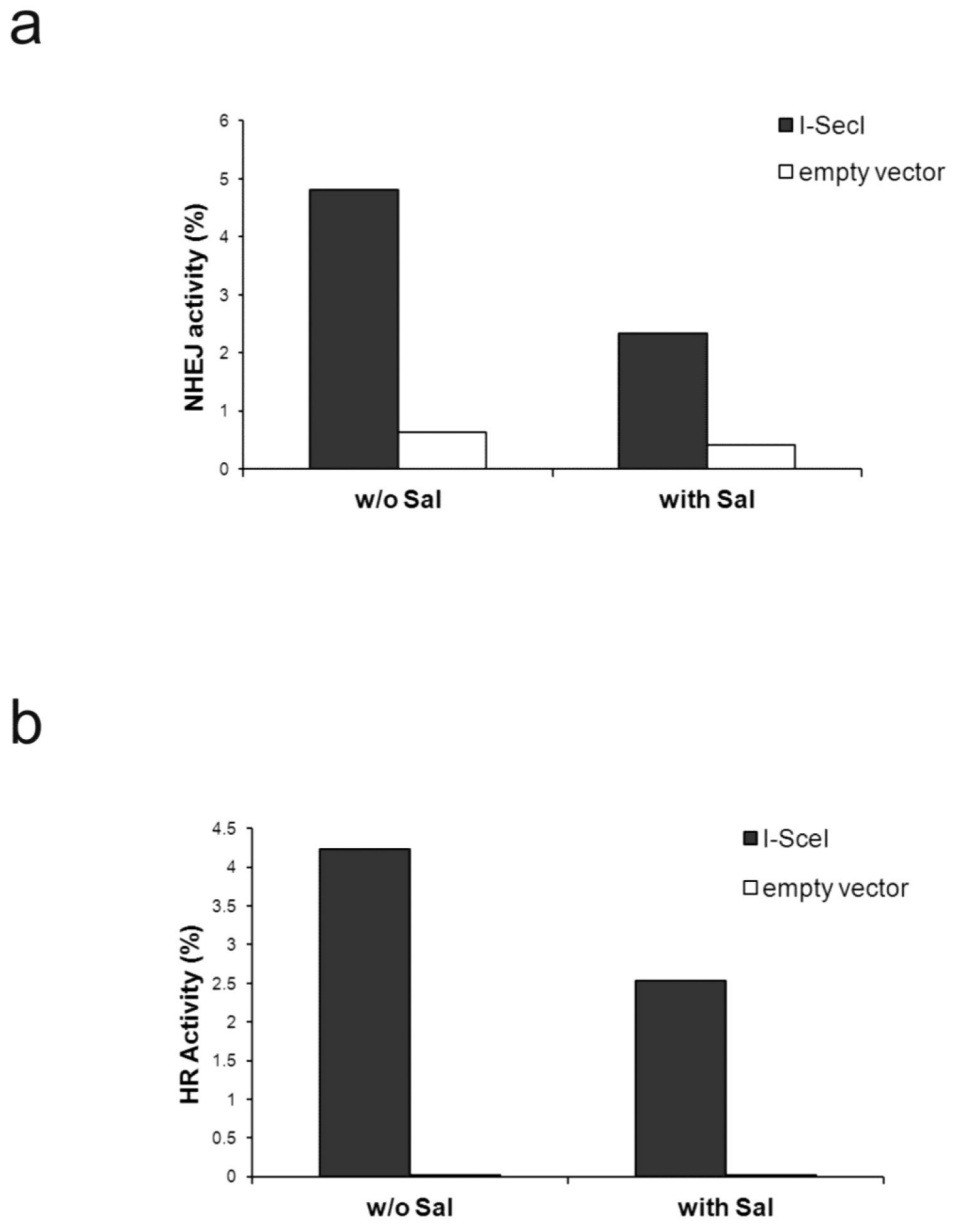
Salubrinal inhibits NHEJ and HR repair: **a**. Measurement of NHEJ activity was conducted 24 hours post-transfection with I-SceI. The experiment was reproduced once with similar results i.e. 4% repair activity for control and 3% for salubrinal treated cells. **b**. Measurement of HR activity was conducted 36 hours post-transfection with I-SceI. The experiment was reproduced once with similar results i.e. 2.35% activity for control and 1.6% activity for salubrinal treated cells at 24 hours post-transfection with I-SceI. Sal - 4.5 µM salubrinal.

Radiation led to increased level of BRCA1, a protein that participates in DNA repair following radiation damage [41]. The increase in BRCA1 was abrogated by salubrinal and by transient expression of the phosphomimetic eIF2α S51D suggesting that excessive eIF2α phosphorylation modulates DNA damage repair (Figure 7). Indeed experiments with HeLa cells and U2OS cells expressing reporter plasmids for NHEJ and HRR respectively showed that salubrinal inhibited repair of I-SceI-induced DSB via both mechanisms (Figure 8). 

### Increased and sustained phosphorylation affects response of breast cancer cells to Vorinostat

**Figure 9:**
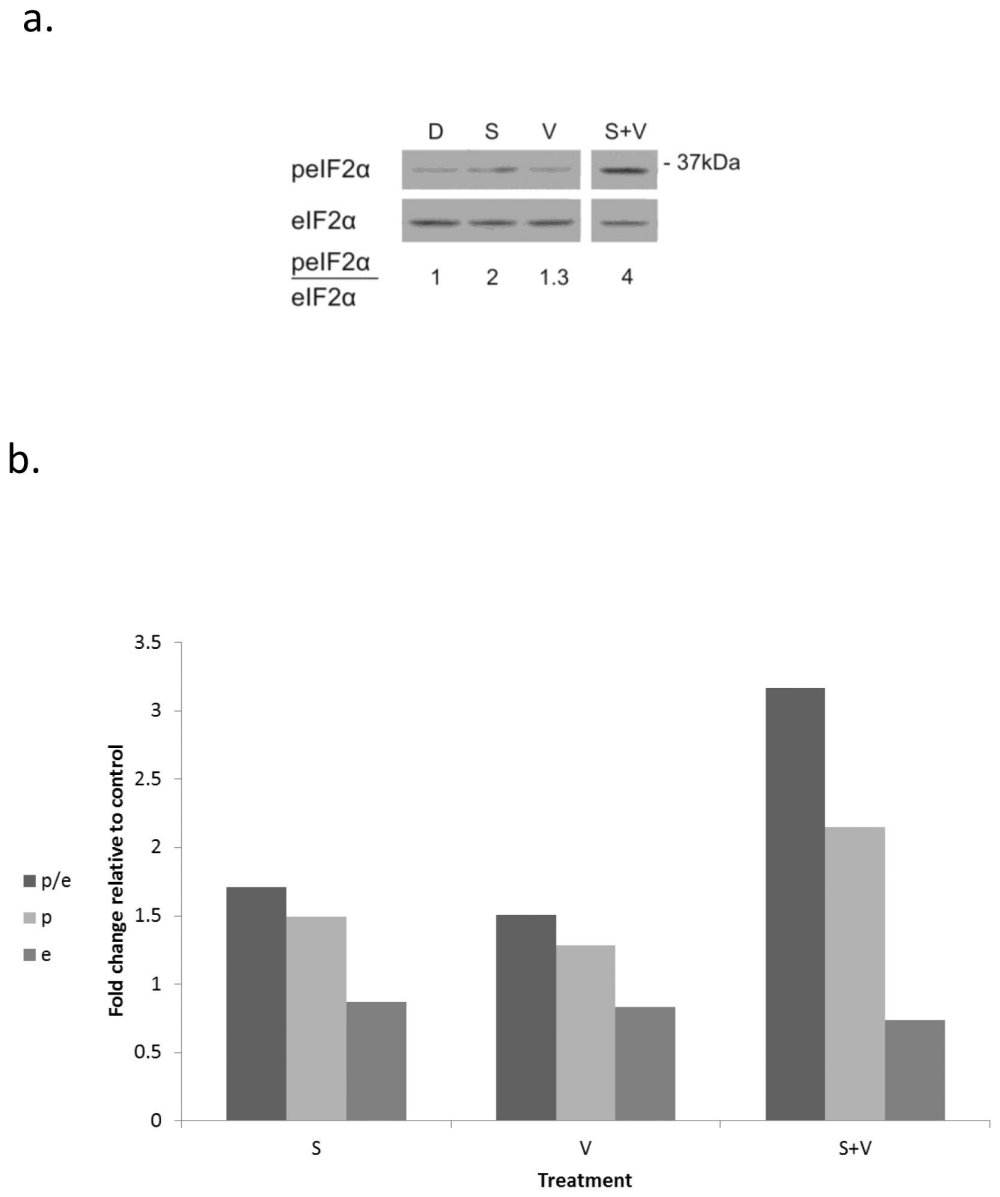
Combined treatment of salubrinal and Vorinostat increases eIF2α phosphorylation: **a**. Cells were harvested 48 hours following application of the vehicle (D), 4.5 µM salubrinal (S), 0.75 µM Vorinostat (V) or both (V+S) and processed for Western blot analysis of eIF2α phosphorylation. **b**. Mean fold change relative to control of eIF2α (e), peIF2α (p) and of the ratio peIF2α/eIF2α (p/e). The experiment was reproduced once with similar results: (Values of e, p, and p/e from both experiments are presented in Table S5 in File S1).

Similar to radiation, the HDACi – Vorinostat also leads to increased eIF2α phosphorylation. This increase has been thought of as a mean of cellular protection against damage [26]. However combining low concentrations of salubrinal and Vorinostat, at concentrations that individually hardly affect eIF2α phosphorylation, resulted in increased phosphorylation of eIF2α, which once again was associated with increased clonogenic death (Figure 9, Table 4, Table S5 in File S1). 

**Table 4:** Combined treatment of salubrinal and Vorinostat enhanced clonogenic cell death.

**Control IF (%)**	**Sal IF (%)**	**V IF (%)**	**Sal+V IF (%)**	**Calculated Additive**
0±1.5	10±0.7	54±1	80±0.3	59

Cells plated for clonogenic survival assays were treated with 4.5 µM salubrinal (Sal), 0.75 µM Vorinostat (V) or both. Numbers are means IF (%) of triplicate samples ± SEM. Theoretical additive effect of salubrinal and Vorinostat on the size of killed cellular fraction is presented under 'calculated additive'. Differences among all experimental groups as well as between each experimental group and control were significant *p*<0.05.

### Specific mTOR inhibitor leads to increased eIF2α phosphorylation in rapamycin resistant breast cancer cells

**Figure 10:**
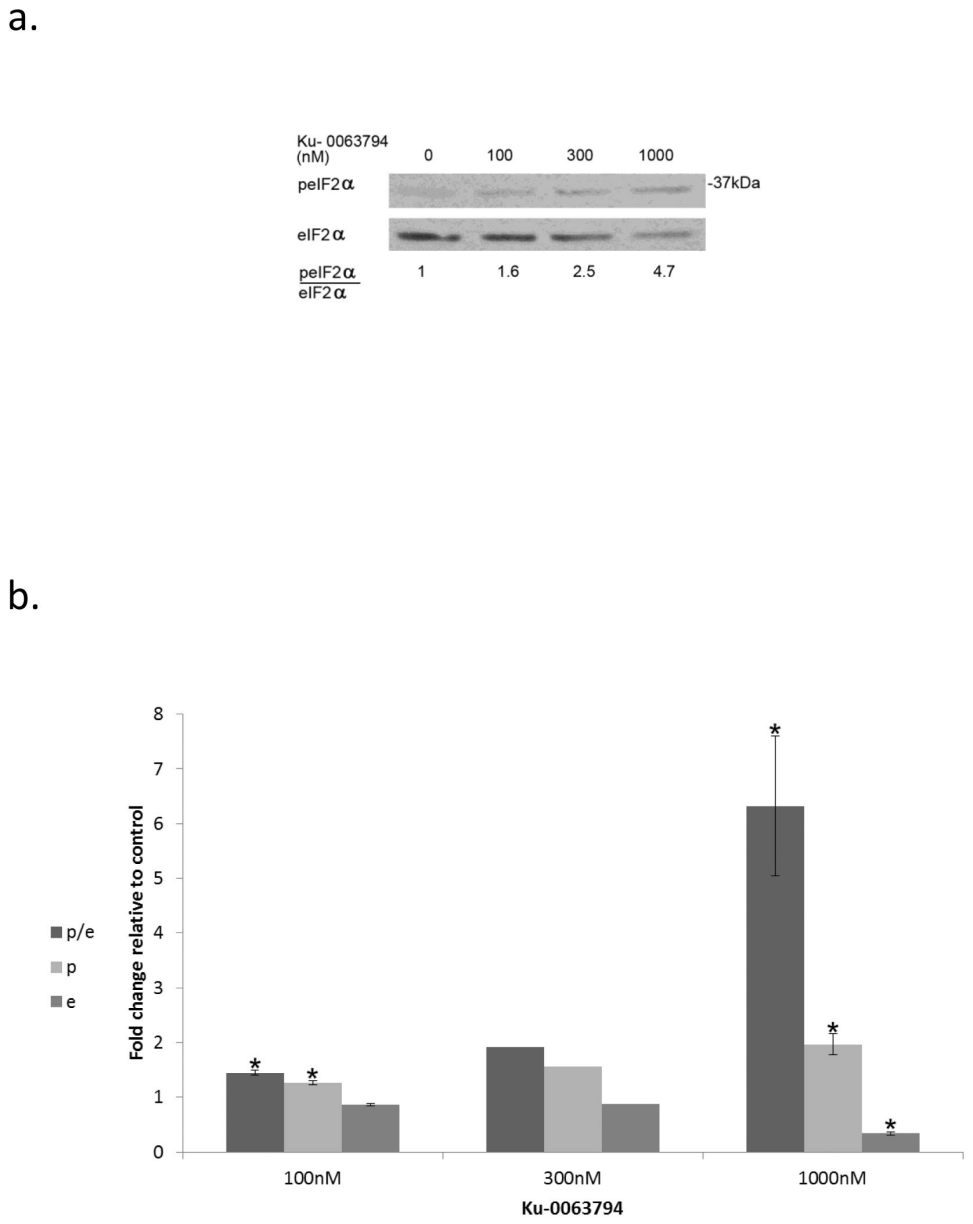
Catalytic inhibitor of mTOR increases eIF2alpha phosphorylation: **a**. Cells were treated with the noted concentrations of Ku-0063794 for 48 hours, then harvested and processed for Western blot analysis of eIF2α phosphorylation. **b**. Ku-0063794 induced changes in eIF2α (e), p-eIF2α (p) and the ratio p-eIF2α/eIF2α (p/e) represent the analysis of 3 determinations for 100 and 1000 nM and of 2 determinations for 300 nM. *Denotes significant change relative to control - *p*<0.05. Values of e, p and p/e for 300 nM Ku-0063794 from the two determinations are presented in Table S6 in File S1).

KU-0063794 is a specific mTOR inhibitor which competes with ATP binding for the catalytic site of the enzyme [42]. Its addition to the rapamycin insensitive MDA-MB-231 cells led to increased eIF2α phosphorylation coupled with decreased clonogenic survival, showing that indeed mTOR activity can modulate that of eIF2α (Figure 10, Table 5,Table S6 in File S1). 

**Table 5:** mTOR catalytic inhibitor - Ku-0063794 - induces clonogenic death.

**Ku (nM)**	**0**	**100**	**300**	**1000**
**IF(%)**	0±1	29±4	55±1	100±0

Cells plated for clonogenic survival assays were treated with increasing concentrations of Ku-0063794 (Ku). Numbers are means IF (%) of triplicate samples ± SEM. Differences among all experimental groups as well as between each experimental group and control were significant *p*<0.05.

## Discussion

Our previous finding that inhibition of mTOR increased sensitivity of MCF-7 cells to ionizing radiation [27], prompted us to search for rapamycin downstream targets that mediate its radiosensitizing effect. Even though several studies have reached the conclusions that phosphorylation of eIF2α during autophagy [15] and hypoxia [11]lies either upstream or parallel to the rapamycin sensitive pathway, we took special note of the work by Kubota et al., who showed that in *Saccharomyces cerevisiae* rapamycin increases eIF2α phosphorylation via activation of GCN2 [10]. While this work was in progress, later studies showed that temsirolimous at concentrations higher than 10 µM can induce eIF2α phosphorylation in temsirolimous-resistant cells [14], and nM concentrations of rapamycin induced eIF2α phosphorylation in acute myeloid leukemia cells [13]. 

Nonetheless, very recently Mounir et al. demonstrated that inhibition of PI3K in MEF leads to inhibition of Akt with the consequent activation of PERK and phosphorylation of eIF2α [12]. Relevant to these findings is an earlier work by Sarbassov et al. [8], who showed that long incubations with rapamycin can destabilize mTORC2 leading to inactivation of Akt. Taken together, these two reports suggest that inhibition of mTOR could potentially lead to increased phosphorylation of eIF2α. However, Mounir et al. and Thoreen et al. who treated U87 cells or MEF with Ku-0063794 - a specific catalytic inhibitor of mTORC1 and mTORC2 - concluded categorically that inhibition of mTOR does not affect phosphorylation of eIF2α [12,16]. 

It remains to be determined if the phenomenon we demonstrated in our study e.g. increased eIF2α phosphorylation following inhibition of mTOR by rapalogues or by Ku-0063794, results from inactivation of mTORC1, mTORC2 – Akt pathway, or whether additional mTOR mediated pathways are involved. We have however clearly shown that prolonged incubation of cancer cells with mTOR inhibitors can lead to sustained elevation of eIF2α phosphorylation. 

It is important to note though that deregulation of mTOR leads to ER overload and to increased phosphorylation of eIF2α [24], showing that either deregulated increase in mTOR activity or its inhibition leads to similar effect on eIF2α phosphorylation. Whether or not both phenomenon are regulated by the same kinases remains to be determined. 

A chemical screen for compounds that protect cells against ER stress has led to the discovery of Salubrinal – an inhibitor of eIF2α dephosphorylation [20]. Salubrinal protected PC12 cells against tunicamycin-induced ER stress but enhanced ER stress in pancreatic β cells exposed to fatty acid, and it has been suggested that the final physiological outcome of increased eIF2α phosphorylation is dependent upon the type of stress signal and the kinase that relays it, the duration of the signal and the type of cells involved in the process [21]. 

Enhanced phosphorylation of eIF2α following exposure to salubrinal can alleviate ER stress caused mainly by accumulation of unfolded proteins in the ER. However, in the case of fatty acids, excessive and sustained phosphorylation of eIF2α may aggravate cell damage by interfering with activation of stress response and consequently with fatty acid oxidation thus leading to accumulation of lipid droplets and to enhancement of the initial damage [43,44] . 

MEF homozygous for eIF2α A/A have also been employed in many studies to demonstrate the role that eIF2α phosphorylation plays in the regulation of other pathways and in cell survival [26]. Decreased survival of these cells is taken as a proof for the protective role played by eIF2α phosphorylation. However, this interpretation does not take into account the possibility mentioned above that while a regulated phosphorylation may be protective its total abrogation as well as its excessive manifestation may be equally harmful. 

Our experiments with salubrinal and with the eIF2α variants show that sustained increase in eIF2α phosphorylation is detrimental to the cells' survival. We also showed that level of eIF2α phosphorylation that is tolerated by non-irradiated cells is harmful in irradiated ones. The fact that treatment with salubrinal decreases expression of surface ESA, suggests that increasing eIF2α phosphorylation is likely to affect the self renewal capacity of the cells. 

Of interest is the finding that enhancing eIF2α phosphorylation in irradiated cells abrogates the radiation-induced increase in BRCA1 level. It is quite possible that the translational machinery is damaged in irradiated cells in more than one way and that increasing eIF2α phosphorylation beyond a certain level further hinders its activity; it is also possible that excessive eIF2α phosphorylation in the presence of radiation-induced eIF2α kinase(s) leads to proteasomal degradation of BRCA1 and possibly of other DNA repair proteins. This hypothesis is in line with the recent findings of Raven et al. who demonstrated that increased PKR or PERK activity in the presence of an increased eIF2α phosphorylation leads to proteasomal degradation of cyclin D1 [45]. 

While this work was in progress Kim et al. showed that in cells that do not express caspase-3, radiation increased ER stress leading to activation of PERK and increased phosphorylation of eIF2α, autophagy and sensitivity to radiation [46]. We have previously shown that similar to rapamycin radiation leads to inhibition of mTORC1 functions [27]. It remains to be determined if under our experimental conditions both radiation and rapamycin also inactivate mTORC2 functions thus leading to activation of PERK and possibly of other eIF2α kinases, or whether additional pathways are involved in this process. 

Finally, to the best of our knowledge, our experiments connect, for the first time, excessive phosphorylation of eIF2α during genotoxic stress with inhibition of DNA repair. Pertinent to our experiments is a recent study by Chen et al. who demonstrated that rapamycin inhibits both homologous and non-homologous end joining DNA repair in MCF-7 cells [47]. Importantly, the fact that sustained and excessive phosphorylation of eIF2α interferes with DNA repair implicates continuous stress signals such as exposure to toxins or sustained ER load with genetic instability.

In conclusion we have demonstrated that eIF2α is a downstream effector of the mTOR pathway, and that excessive phosphorylation of eIF2α interferes with DNA repair processes and negatively affects survival of cancer cells. Our results suggest that targeting eIF2α will both potentiate the effects of established anti-neoplastic therapies and help circumvent resistance to rapalogues.

## Supporting Information

File S1 **Tables S1, S2, S3, S4, S5 and S6.**
(DOC)

Figure S1 **eIF2α variants do not alter radiation-induced phosphorylation of endogenous eIF2α.** Cells were transfected with plasmids expressing non-phosphorylatable eIF2α S51A (SA) or the phosphomimetic eIF2α S51D (SD) and processed for analysis of eIF2α phosphorylation 48 hours post-irradiation.
(TIF)
