# Supplementary material for: Eukaryotic Initiation Factor 2α - a Downstream Effector of Mammalian Target of Rapamycin - Modulates DNA Repair and Cancer Response to Treatment
Source: PLoS One. 2013 Oct 25;8(10):e77260. doi: 10.1371/journal.pone.0077260 (PMC3808413; doi:10.1371/journal.pone.0077260)
Supplement: File S1 — Tables S1, S2, S3, S4, S5 and S6. (DOC) [file pone.0077260.s001.doc]

**Table S1. Salubrinal does not alter cell cycle distribution of U2OS**

| **Treatment** | **G0/G1** | **S** | **G2+M** | **Sub G1** |
| --- | --- | --- | --- | --- |
| **DMSO** | 61.2 | 14.3 | 21.7 | 0.8 |
| **Sal (4.5 µM)** | 59.8 | 16.3 | 20.9 | 0.4 |

Cell cycle distribution was determined in propidium iodide stained cells 24 hours following addition of 4.5 µM salubrinal to experimental and DMSO to control.

**Table S2. MCF-7 cells are more sensitive to rapamycin treatment than MDA-MB-231**

| **Cells** | **Rapamycin (µM)** | **IF (%)** |
| --- | --- | --- |
| **MCF-7** | 0 | 0±2 |
|  | 0.050 | 45±3 |
| **MDA** | 0 | 0±1.3 |
|  | 1 | 8±5 |
|  | 2 | 11±2.1 |

Cells were plated and processed for colony survival assay. Numbers are IF (%) ± SEM of triplicate samples. Differences between control and experimental groups were significant (*p*<0.05) only for MCF-7 cells.

**Table S3**. **Salubrinal enhances radiation induced eIF2α phosphorylation**

| **Treatment** | **EXP#** | **p** | **e** | **p/e** |
| --- | --- | --- | --- | --- |
| 1.5 Gy | 1 | 1.5 | 0.94 | 1.6 |
|  | 2 | 1.45 | 1.22 | 1.2 |
|  | **Average** | **1.475** | **1.08** | **1.4** |
|  |  |  |  |  |
| 9.5 Gy | 1 | 1.9 | 0.84 | 2.2 |
|  | 2 | 2.2 | 1.17 | 1.9 |
|  | **Average** | **2.05** | **1.005** | **2.05** |
|  |  |  |  |  |
| Sal | 1 | 3 | 1 | 3 |
|  | 2 | 1.8 | 0.95 | 1.9 |
|  | **Average** | **2.4** | **0.975** | **2.45** |
|  |  |  |  |  |
| 1.5 Gy + Sal | 1 | 3 | 0.78 | 4 |
|  | 2 | 1.43 | 0.69 | 2.1 |
|  | **Average** | **2.215** | **0.735** | **3.05** |
|  |  |  |  |  |
| 9.5 Gy + Sal | 1 | 4.5 | 0.35 | 1.4 |
|  | 2 | 1.9 | 0.54 | 3.4 |
|  | **Average** | **3.2** | **0.445** | **8.7** |

Cells were irradiated with the noted radiation dose prior to addition of 4.5 µM Salubrinal (Sal). Numbers are densitometry derived values from two separate experiments for fold increase in eIF2α (e), p-eIF2α (p) and the ratio p-eIF2α/eIF2α (p/e) in treated cells relative to control.

**Table S4.** **Salubrinal enhances clonogenic death in irradiated** **MCF-7**

| **Rads** | **-Sal IF (%)** | **+Sal IF (%)** | **Calculated additive** |
| --- | --- | --- | --- |
| 0 | 0±2.3 | 29±1.3 |  |
| 85 | 35±1.6 | 66±1 | 54 |

Cells were plated and processed for colony survival assay. Salubrinal (3µM) was added to the cells immediately following irradiation. Numbers are IF (%) ± SEM of triplicate samples. Differences between each experimental group and control as well as between cells receiving both treatments to groups receiving one treatment were significant (*p*<0.05)

**Table S5. Combined treatment of salubrinal and Vorinostat increases eIF2α phosphorylation**

| **Treatment** | **Experiment #** | **p** | **e** | **p/e** |
| --- | --- | --- | --- | --- |
| **S** | 1 | 1.65 | 0.8 | 2 |
|  | 2 | 1.34 | 0.94 | 1.42 |
|  | **Average** | **1.495** | **0.87** | **1.71** |
|  |  |  |  |  |
| **V** | 1 | 1 | 0.76 | 1.3 |
|  | 2 | 1.57 | 0.9 | 1.72 |
|  | **Average** | **1.285** | **0.83** | **1.51** |
|  |  |  |  |  |
| **S+V** | 1 | 2.1 | 0.53 | 4 |
|  | 2 | 2.2 | 0.94 | 2.33 |
|  | **Average** | **2.15** | **0.735** | **3.165** |

Cells were harvested 48 hours following application of the vehicle, 4.5 µM salubrinal (S), 0.75 µM Vorinostat (V) or both (V+S) and processed for Western blot analysis of eIF2α phosphorylation. Numbers are densitometry derived values from two separate experiments for fold increase in eIF2α (e), p-eIF2α (p) and the ratio p-eIF2α/eIF2α (p/e) in treated cells relative to control.

**Table S6. Ku-0063794 increases eIF2α phosphorylation**

| **Treatment** | **Experiment #** | **p** | **e** | **p/e** |
| --- | --- | --- | --- | --- |
| **300 nM Ku 0063794** | 1 | 1.64 | 0.65 | 2.5 |
|  | 2 | 1.49 | 1.1 | 1.35 |
|  | **Average** | **1.565** | **0.875** | **1.925** |

Cells were treated with 300 nM of Ku-0063794 for 48 hours, then harvested and processed for Western blot analysis of eIF2α phosphorylation. Numbers are densitometry derived values from two separate experiments for fold increase in eIF2α (e), p-eIF2α (p) and the ratio p-eIF2α/eIF2α (p/e) in cells treated with the inhibitor relative to control.
