# Supplementary figures and images for: Eukaryotic Initiation Factor 2α - a Downstream Effector of Mammalian Target of Rapamycin - Modulates DNA Repair and Cancer Response to Treatment
Source: PLoS One. 2013 Oct 25;8(10):e77260. doi: 10.1371/journal.pone.0077260 (PMC3808413; doi:10.1371/journal.pone.0077260)

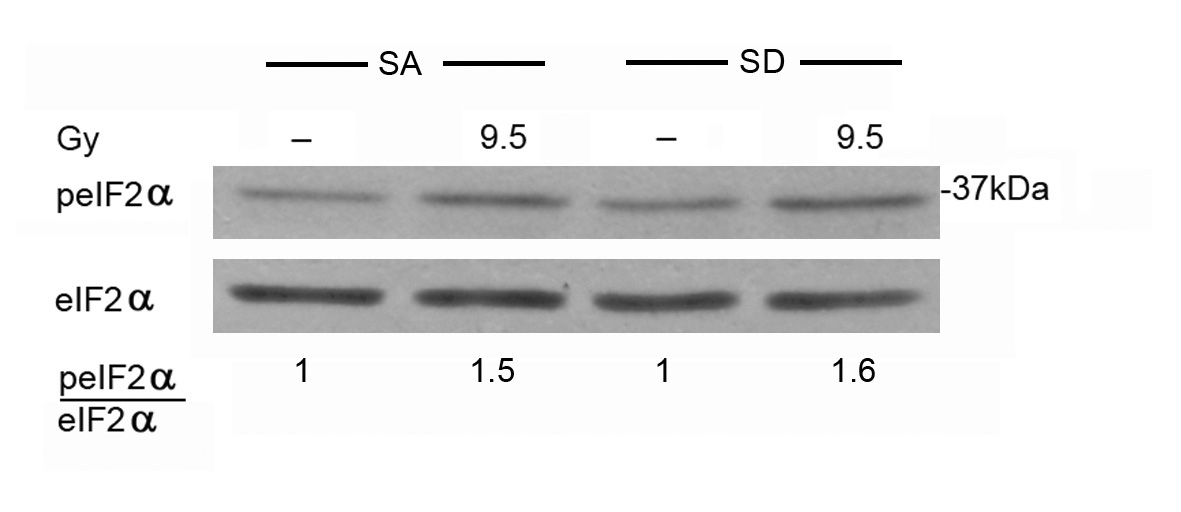

Supplement: Figure S1 — eIF2α variants do not alter radiation-induced phosphorylation of endogenous eIF2α. Cells were transfected with plasmids expressing non-phosphorylatable eIF2α S51A (SA) or the phosphomimetic eIF2α S51D (SD) and processed for analysis of eIF2α phosphorylation 48 hours post-irradiation. (TIF) [file pone.0077260.s002.tif]
